# Endometriosis and Chronic Endometritis: Shared Mechanisms, Diagnostic Challenges, and Clinical Implications in Infertility

**DOI:** 10.3390/diagnostics16111648

**Published:** 2026-05-27

**Authors:** Siqi Bai, Zihan Zhou, Hong Zhan

**Affiliations:** 1 Department of Obstetrics and Gynecology, Zhoushan Women and Children Hospital, Zhoushan 316000, China; baisiqi_1010@163.com; 2 Zhejiang University School of Medicine, Hangzhou 310000, China; 22418747@zju.edu.cn; 3 Day Surgery Center, Women’s Hospital, Zhejiang University School of Medicine, Hangzhou 310000, China; 4 Zhejiang Provincial Key Laboratory of Precision Diagnosis and Therapy for Major Gynecological Diseases, Women’s Hospital, Zhejiang University School of Medicine, Hangzhou 310000, China

**Keywords:** endometriosis, chronic endometritis, infertility, endometrial receptivity, chronic inflammation, immune dysregulation, implantation failure, diagnosis

## Abstract

Endometriosis (EM) and chronic endometritis (CE) are both implicated in female infertility, yet the relationship between them remains incompletely understood. In this narrative review, we synthesize non-systematically selected clinical and mechanistic evidence on EM–CE coexistence, with emphasis on infertility-related settings and diagnostic uncertainty. Available studies, largely from selected infertility cohorts, suggest that CE is identified more often in women with EM, raising the possibility that their coexistence reflects a biologically meaningful association rather than incidental overlap. The two conditions share several abnormalities that may impair reproduction, including persistent inflammation, immune dysregulation, altered cytokine and chemokine signaling, impaired macrophage and natural killer cell function, progesterone resistance, and reduced endometrial receptivity. In addition to the pelvic anatomical distortion and ovarian dysfunction associated with EM, CE may further compromise implantation by impairing decidualization, displacing the window of implantation, and disrupting the local endometrial microenvironment. Both conditions also remain diagnostically challenging. EM is most reliably confirmed by laparoscopy with histologic verification, whereas CE is generally diagnosed by hysteroscopy and endometrial biopsy demonstrating plasma cells. For CE in particular, the lack of standardized diagnostic criteria and uniform CD138 thresholds continues to limit diagnostic consistency. Emerging imaging techniques, molecular biomarkers, microbiota-based approaches, and artificial intelligence-assisted models remain investigational or adjunctive rather than established tools for routine integrated assessment. Taken together, the current evidence is hypothesis-generating and supports selective, phenotype-driven consideration of coexisting EM and CE in infertility care rather than routine dual invasive evaluation. Further studies are required to clarify the mechanisms linking these conditions, define their combined reproductive impact, and determine whether integrated diagnostic and therapeutic strategies can improve fertility outcomes.

## 1. Introduction

Endometriosis (EM) is an estrogen-dependent chronic inflammatory condition in which endometrium-like tissue is found outside the uterine cavity. It is estimated to affect about 10% of women of reproductive age [1,2,3,4] and its prevalence rises substantially among women evaluated for infertility, in whom rates of 25–50% have been reported [2,5]. Clinically, EM is not only a major cause of chronic pelvic pain and diminished quality of life, but also a well-recognized contributor to subfertility. Its adverse reproductive effects are multifactorial and may involve distortion of pelvic anatomy, abnormalities in the follicular and peritoneal milieu, impaired progesterone responsiveness, and reduced endometrial receptivity [6]. Although imaging techniques and surgical management have improved, timely diagnosis remains difficult in many patients because symptoms vary widely and currently available diagnostic methods still have important limitations [7].

Chronic endometritis (CE) is a chronic inflammatory condition of the endometrium in which plasma cells are identified within the endometrial stroma on histologic examination [8]. The condition is often low grade, and its clinical presentation can be minimal or entirely absent. While CE does not appear to be particularly common in the general gynecologic population, it has been observed more frequently in women with infertility, recurrent implantation failure (RIF), and recurrent pregnancy loss (RPL) [9,10]. This pattern has increased interest in its reproductive significance. Diagnosis, however, is still problematic because hysteroscopic findings are nonspecific [11], histopathologic criteria vary between studies, and no universally accepted threshold for CD138-positive plasma cells has been established.

The possible association between EM and CE has received increasing attention [9,12,13]. One reason is that both disorders share several features relevant to infertility, including persistent inflammation, immune imbalance, and impaired endometrial function. Their coexistence may therefore have particular clinical relevance in selected women with infertility. However, recent reviews have mainly emphasized biological overlap and descriptive coexistence; less attention has been paid to how the available evidence should be weighed, which patient subgroups may warrant selective dual evaluation, and where current diagnostic claims remain insufficiently supported. This narrative review therefore focuses on the clinical and diagnostic interpretation of the EM–CE relationship in infertility, while explicitly avoiding claims of systematic completeness or quantitative certainty.

## 2. Methods and Scope of the Narrative Review

This article was conducted as a narrative review rather than a systematic review or meta-analysis. We searched PubMed/MEDLINE, Web of Science, Embase, and Google Scholar for relevant publications up to April 2026, using terms related to endometriosis, chronic endometritis, infertility, endometrial receptivity, and diagnosis. Reference lists of relevant reviews, guidelines, and key original studies were also screened.

We included clinical studies, systematic reviews, meta-analyses, guidelines, and selected mechanistic studies relevant to the association between endometriosis and chronic endometritis, their shared inflammatory and endometrial mechanisms, diagnostic challenges, and infertility-related implications. Because of the narrative design, no formal risk-of-bias assessment or quantitative pooling was performed. Evidence was interpreted according to study design, clinical relevance, diagnostic clarity, and consistency with the broader literature.

## 3. Epidemiological Association Between Endometriosis and Chronic Endometritis

Observational studies indicate that CE is identified more frequently in women with EM, particularly in infertility-centered populations [14,15,16]. Conversely, a substantial proportion of women diagnosed with CE have also been reported to have coexisting EM [14,15]. These findings support an epidemiological association between the two conditions, although the strength of that association remains uncertain. Because the available direct coexistence studies are few and heterogeneous, we summarized reported patterns rather than pooling prevalence estimates or measures of association. Interpretation of this association is limited by substantial heterogeneity in study design, patient selection, and diagnostic criteria. Most available studies are retrospective or cross-sectional and are based largely on infertility cohorts, which may overrepresent both conditions relative to the general gynecologic population [17,18]. Reported coexistence rates may therefore be influenced by referral bias, selection bias, reproductive phenotype, and incompletely controlled confounders, including age, prior hormonal treatment, pelvic surgery, and assisted reproductive technology (ART) exposure.

One major source of inconsistency lies in the diagnosis of CE. In contrast to the increasing standardization of EM classification and imaging assessment, CE still lacks universally accepted diagnostic thresholds [9,11]. Although endometrial biopsy with CD138 immunohistochemical staining is widely used, the threshold for a positive diagnosis varies considerably across studies [9]. In addition, focal plasma cell infiltration, biopsy timing, specimen quality, and histopathological interpretation may all affect diagnostic yield [9,11]. Interpretation is further complicated by the heterogeneity of EM itself, as superficial peritoneal lesions, ovarian endometriomas, and deep infiltrating disease may differ in inflammatory burden and reproductive impact [2,4,19,20]. Another limitation of the current evidence is that CE prevalence is rarely analyzed according to EM subtype or disease stage.

The nature of the relationship between the two conditions is also uncertain. CE may directly contribute to an endometrial milieu that is unfavorable for reproduction, whereas EM itself may perpetuate chronic endometrial inflammation through sustained inflammatory signaling and disturbed uterine immune homeostasis [9,12,14,15]. It is also possible that EM and CE develop in the same susceptible population because they share overlapping inflammatory, hormonal, and immune disturbances. Therefore, coexistence should currently be interpreted as a clinically relevant but incompletely quantified association rather than evidence of a single causal pathway. Taken together, the published data point to a clinically meaningful association between EM and CE, particularly in selected infertility populations, but firm conclusions are limited by marked heterogeneity and the likelihood of selection bias [14,15]. Relevant studies are presented in Table 1 according to evidence category so that direct coexistence data can be distinguished from mechanistic support and interpretive background.

## 4. Shared Pathophysiological Mechanisms

**Figure 1:**
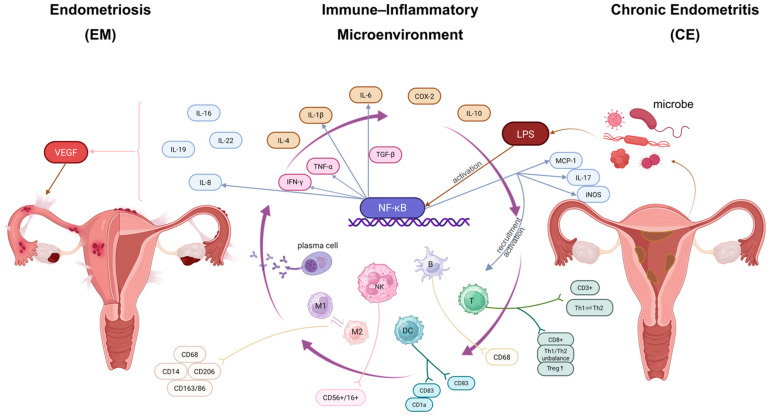
Schematic overview of the shared immune–inflammatory microenvironment in endometriosis (EM) and chronic endometritis (CE). Although EM and CE arise in different anatomical compartments, both involve persistent inflammatory signaling, altered immune-cell composition and function, abnormal cytokine and chemokine expression, hormonal dysregulation, and impaired endometrial homeostasis. These overlapping alterations may contribute to disease coexistence, impaired endometrial receptivity, and infertility. Abbreviations: CE, chronic endometritis; COX-2, cyclooxygenase-2; CXCL13, C-X-C motif chemokine ligand 13; DC, dendritic cell; EM, endometriosis; ER, endoplasmic reticulum; IKK, IκB kinase; IL, interleukin; iNOS, inducible nitric oxide synthase; LPS, lipopolysaccharide; MMP, matrix metalloproteinase; NF-κB, nuclear factor kappa-B; NK, natural killer; NLRP3, NLR family pyrin domain-containing 3; TLR4, Toll-like receptor 4; TNF-α, tumor necrosis factor-alpha; VEGF, vascular endothelial growth factor.

Although EM and CE are different diseases, they share selected biological disturbances that may be relevant to infertility, particularly at the level of the endometrial immune microenvironment and receptivity [12,15,26,27]. Their origins differ, but downstream pathways involving inflammation, immune regulation, hormonal responsiveness, and endometrial function appear to overlap. In EM, these changes are tied mainly to ectopic lesion survival and an altered pelvic microenvironment; in CE, they are more directly related to ongoing intrauterine inflammation and loss of endometrial homeostasis [9,12]. Among the shared domains discussed below, evidence is most consistent for chronic inflammatory signaling, immune-cell disturbance, and impaired endometrial receptivity; microbiota-related mechanisms and extra-endometrial interactions remain more exploratory. The main shared biological pathways are shown in Figure 1.

### 4.1. Chronic Inflammatory Signaling

Persistent inflammatory signaling is a common feature of both EM and CE. Key mediators include IL-1β, IL-6, IL-8, and TNF-α, together with downstream activation of NF-κB and related inflammatory pathways [28,29,30,31,32,33,34,35,36]. In EM, IL-6, IL-1β, and TNF-α are involved in lesion establishment, stromal-cell proliferation, immune escape, angiogenesis, and tissue remodeling [28,30,31,32,33,37,38,39]. In CE, a similar proinflammatory milieu is evident, with increased IL-6, IL-1β, and TNF-α detected in menstrual effluent and endometrial samples [34,35]. Experimental studies further suggest that microbial inflammatory stimuli may activate NF-κB signaling and sustain the expression of mediators involved in chronic endometrial injury and repair [29,35,36,40,41]. Taken as a whole, both conditions appear to be sustained by persistent inflammatory activation, although their upstream triggers are likely different.

### 4.2. Immune-Cell Dysfunction

The normal endometrium contains a dynamic immune-cell network that contributes not only to host defense but also to cyclical remodeling, decidualization, vascular adaptation, and embryo implantation [10,42,43,44,45]. In both EM and CE, this immune balance appears to be disturbed. In EM, reduced NK-cell cytotoxicity, increased macrophage infiltration, and abnormalities in dendritic cells, B cells, and T-cell subsets contribute to a dysregulated immune microenvironment that favors lesion persistence [46,47,48,49,50,51,52,53,54,55,56,57,58,59]. In CE, local immune changes have been described at multiple levels, including increased immunoglobulin expression, alterations in NK-cell subsets, expansion of macrophage and dendritic-cell populations, and a shift in T-helper-cell balance toward a proinflammatory state [10,60,61,62,63]. Although the specific abnormalities are not identical, both conditions show disturbed immune-cell composition and function, which may impair endometrial receptivity and tissue homeostasis.

### 4.3. Hormonal Dysregulation and Defective Decidualization

Hormonal dysregulation and defective decidualization represent another key area of overlap between EM and CE. In EM, progesterone resistance is a major pathogenic feature that may impair endometrial decidualization, while estrogen-dependent activity is often enhanced [64,65]. Epigenetic alterations have also been reported and may further contribute to abnormal endometrial responsiveness [64,66]. In CE, persistent inflammation may likewise interfere with hormone-dependent maturation of the endometrium, leading to impaired decidualization, displacement of the window of implantation, and abnormal uterine perfusion [11]. Increased estrogen receptor expression has also been reported in CE and may delay progesterone-driven transformation of the endometrium [11]. Although the underlying mechanisms are not identical, both EM and CE may disrupt the endocrine and cellular processes required for implantation.

### 4.4. Microbiota and the Mucosal Immune Interface

The reproductive tract microbiota and mucosal immune environment may represent another area of overlap, although the evidence is stronger for CE than for EM. In CE, microbial persistence and LPS-related inflammation are thought to contribute directly to persistent endometrial inflammatory activation [35,36]. Reduced levels of Bifidobacterium and other lactic-acid-producing bacteria have been reported in women with infertility and CE, and elevated endometrial LPS levels correlate with IL-6 expression [35,67]. In EM, dysbiosis and mucosal immune disturbance have been proposed as contributors to persistent inflammation, but the current evidence is limited and less consistent [68,69,70]. Microbiota-related mechanisms should therefore be regarded as a possible rather than established link between the two conditions.

Taken together, these overlapping mechanisms provide a biologically plausible basis for EM–CE coexistence, although their relative contributions to infertility remain incompletely defined. The main shared pathophysiological features are summarized in Table 2.

## 5. Diagnostic Challenges in Suspected Endometriosis–Chronic Endometritis Comorbidity

Diagnosing EM and CE is challenging even when each condition is considered separately, and the difficulty increases when both are present in the same patient. EM and CE involve different anatomical compartments, often present with nonspecific or overlapping features, and follow different diagnostic pathways. Coexisting diseases may therefore be overlooked during infertility evaluation, especially when reproductive failure is attributed to a single diagnosis.

### 5.1. Current Diagnostic Approaches for Endometriosis

The diagnosis of EM is particularly challenging in women with infertility. Current diagnostic evaluation relies mainly on transvaginal ultrasound (TVUS) and pelvic magnetic resonance imaging (MRI), which are most useful for detecting ovarian endometriomas and deep infiltrating EM [71]. However, superficial peritoneal EM is often poorly visualized with conventional imaging. Although laparoscopy with histological confirmation remains the reference standard, its invasive nature and limited role in routine infertility evaluation restrict its use as a first-line diagnostic approach [72]. Even with improved imaging, some clinically meaningful EM lesions remain difficult to detect, particularly when disease is subtle or confined to limited anatomical sites.

### 5.2. Current Diagnostic Approaches for Chronic Endometritis

Compared with EM, CE is diagnosed with less consistency. In routine practice, assessment usually relies on hysteroscopy together with histologic examination of endometrial biopsy specimens. On hysteroscopy, findings such as hyperemia, stromal edema, and micropolyps may raise suspicion of CE, but none of these features is specific, and their reported diagnostic performance varies substantially between studies [73,74,75,76]. Tissue-based diagnosis also has limitations. Plasma-cell identification, often aided by CD138 immunohistochemistry, is widely used in pathologic evaluation, yet the threshold for a positive diagnosis is not uniform and may be affected by biopsy timing, sampling conditions, and interobserver interpretation [77,78]. For this reason, neither hysteroscopic appearance nor histopathology provides a definitive answer in every clinical setting. Such diagnostic uncertainty can make CE easy to overlook, particularly when it is present alongside another chronic reproductive disorder such as EM.

### 5.3. Why Concurrent Disease May Be Overlooked

Coexisting EM and CE can be missed in clinical practice for several reasons. The two conditions arise in different anatomical sites, their manifestations are often nonspecific, and they are usually investigated along separate diagnostic lines. In most patients, suspicion of EM is driven by symptoms, imaging, and occasionally laparoscopy, whereas CE is more often evaluated by hysteroscopy and endometrial biopsy. Because of this separation, identifying one disorder does not automatically trigger assessment for the other.

The issue is particularly important in infertility care. Pelvic pain or abnormal bleeding may direct attention toward EM alone, while CE may remain silent. On the other hand, persistent reproductive failure in a woman diagnosed with CE does not always lead to further investigation for EM. The limitations of current tests also matter: biopsy may miss focal CE, and laparoscopy offers no view of the uterine cavity. As a result, one diagnosis may be made and the other left unrecognized [16]. Available studies support a more alert diagnostic approach in selected patients, but not enough evidence exists to recommend routine dual invasive evaluation for all infertility populations [79,80].

### 5.4. Emerging Diagnostic Opportunities

A range of newer diagnostic approaches is being explored for EM, CE, and the possibility that both conditions may coexist. In EM, this work includes elastography, refined MRI protocols, PET-based imaging, circulating biomarkers, and microRNA-based models [79,80,81,82,83,84]. In CE, proposed tools include microbiota analysis, inflammatory markers such as lipopolysaccharide and IL-6, and menstrual blood markers including fertility α2-microglobulin [35,67,85]. None of these approaches is currently established for routine clinical use.

There is also growing interest in combining diagnostic modalities. Approaches that incorporate symptoms, imaging findings, hysteroscopy, histopathology, and molecular data may prove more informative than any single test alone [86,87]. Artificial intelligence-based image analysis and predictive models may further improve risk assessment in women suspected of having both conditions [88,89]. Still, prospective studies are needed before these methods can be applied in routine practice. For now, clinicians must continue to rely mainly on clinical phenotype and selective integrated evaluation.

### 5.5. Clinical Implications for Infertility Evaluation

The possible coexistence of EM and CE is most clinically relevant in selected infertility populations, including women with recurrent implantation failure, recurrent pregnancy loss, unexplained infertility, or ongoing reproductive failure despite treatment for known EM. In such patients, it may be more useful to consider the two conditions together rather than pursue them as entirely separate diagnostic entities [12].

Current evidence does not support universal CE screening in all women with EM or routine dual invasive evaluation in all patients with infertility. A more practical approach is selective evaluation based on the clinical phenotype. In practical terms, targeted assessment for CE may be considered in women with EM who have unexplained infertility, recurrent implantation failure, recurrent pregnancy loss, persistent abnormal uterine bleeding, or reproductive failure disproportionate to the pelvic findings. Conversely, further evaluation for EM may be considered in women with CE who also have severe dysmenorrhea, chronic pelvic pain, dyspareunia, adnexal masses, or imaging abnormalities [16,24]. Because no standardized algorithm is currently available to identify which patients are most likely to benefit from dual evaluation, management should be individualized. An additional unresolved issue is whether identification and treatment of CE in women with EM can improve reproductive outcomes beyond those achieved with standard EM-directed management alone; the available evidence remains limited and heterogeneous [23].

The main diagnostic approaches for EM and CE, together with their potential role in identifying coexisting disease, are summarized in Table 3. Based on the current evidence, practical considerations for selective, phenotype-driven evaluation of suspected EM–CE comorbidity are outlined in Box 1.

Box 1 **Clinical practice points for suspected EM–CE comorbidity.**

**When to consider CE assessment in patients with known or suspected EM**

▯ Unexplained RIF or RPL.
▯ Abnormal uterine bleeding that cannot be adequately explained by EM alone.
▯ Persistent infertility despite appropriate EM-directed treatment, either medical or surgical.
▯ Reproductive failure in which pelvic disease burden does not fully account for the clinical phenotype.

**When to consider EM assessment in patients diagnosed with CE**

▯ Severe dysmenorrhea, chronic pelvic pain, dyspareunia, or other symptoms suggestive of EM.
▯ Adnexal masses or imaging findings suggestive of ovarian endometrioma or deep infiltrating EM.
▯ Persistent infertility after CE treatment, particularly when symptoms or imaging findings suggest an additional pelvic factor.

**Practical diagnostic considerations**

▯ In suspected CE, hysteroscopy combined with endometrial biopsy may be considered, particularly in infertility, RIF, or RPL populations. Sampling during the proliferative phase may improve diagnostic interpretation, although practice varies between studies. CD138 immunohistochemistry can improve plasma-cell detection, but diagnostic thresholds remain heterogeneous. A threshold such as ≥5 plasma cells per 10 high-power fields has been proposed in some infertility-focused studies to reduce overdiagnosis, but it should not be regarded as a universally accepted standard.
▯ In suspected EM, transvaginal ultrasound is usually the first-line imaging modality, especially for ovarian endometrioma and some deep infiltrating lesions. MRI may be useful in selected patients with suspected deep disease or complex pelvic anatomy. Laparoscopy should be reserved for selected cases in which confirmation or treatment is expected to influence management.

Note: These points are intended to support selective, phenotype-driven evaluation. They should not be interpreted as recommending routine dual invasive assessment for all women with infertility or universal CE screening in all patients with EM.

## 6. Impact of Coexisting Endometriosis and Chronic Endometritis on Infertility

**Figure 2:**
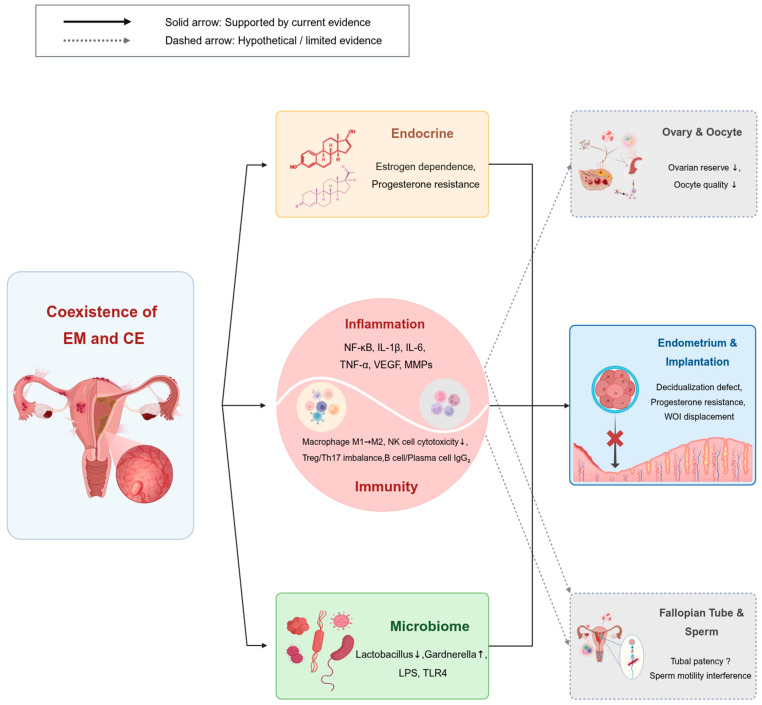
Conceptual framework illustrating potential mechanisms linking coexisting endometriosis (EM) and chronic endometritis (CE) with infertility-related reproductive dysfunction. The figure summarizes the proposed progression from coexisting EM and CE to potential reproductive impairment. EM–CE coexistence is illustrated by multifocal endometriotic lesions, pelvic adhesions, endometrial inflammatory lesions, and endometrial hyperemia. Possible intermediate mechanisms include endocrine dysregulation with estrogen dependence and progesterone resistance, the interaction between immune dysregulation and chronic inflammation, and microbiota-related disturbance. These alterations may contribute to impaired ovarian and oocyte function, reduced endometrial receptivity, and disruption of the tubal–sperm transport pathway. Solid arrows indicate relationships that are relatively better supported in the current literature, whereas dashed arrows represent more speculative or incompletely validated associations. The model is intended as a hypothesis-generating conceptual framework rather than evidence of a direct causal pathway. Abbreviations: CE, chronic endometritis; EM, endometriosis; IL, interleukin; LPS, lipopolysaccharide; MMP, matrix metalloproteinase; NF-κB, nuclear factor kappa-B; NK, natural killer; RPL, recurrent pregnancy loss; TLR4, Toll-like receptor 4; TNF-α, tumor necrosis factor-alpha; VEGF, vascular endothelial growth factor; WOI, window of implantation.

Infertility has been associated with both EM and CE [11]. EM is commonly encountered during infertility work-up, and CE also appears to be more prevalent in women undergoing infertility evaluation, although reported rates differ substantially between studies [92,93]. Because both disorders affect endometrial inflammation, maturation, and receptivity, their coexistence could plausibly add to implantation-related dysfunction, although direct outcome data in confirmed EM–CE comorbidity in selected infertility cohorts remain limited. Figure 2 is therefore presented as a hypothesis-generating conceptual model.

### 6.1. Endometrial Receptivity and Implantation Failure

The endometrium is likely the main site of interaction between EM and CE. In EM, progesterone resistance, altered inflammatory signaling, and abnormal endometrial gene regulation may impair decidualization and implantation [64,65,66]. In CE, persistent endometrial inflammation has been linked to displacement of the window of implantation, defective decidualization, and delayed progesterone-dependent maturation [11]. Reduced expression of genes related to endometrial receptivity and decidualization, including IL11, CCL4, IGF1, PRL, and IGFBP1, has also been reported in CE endometrium [41]. Together, these findings indicate that CE may further aggravate the impaired steroid responsiveness and endometrial dysfunction already present in EM.

Implantation depends on tightly coordinated inflammatory, endocrine, metabolic, and immune processes within the eutopic endometrium [64,94]. However, direct clinical evidence in women with confirmed EM–CE coexistence remains limited; therefore, any additive effect should be regarded as plausible and hypothesis-generating, mainly at the level of endometrial receptivity [11,41,64,95]. EM and CE may also differ in the extent to which they affect fertility beyond implantation.

### 6.2. Broader Reproductive Consequences

The reproductive impact of EM is not limited to implantation. Fertility may also be compromised by pelvic anatomical distortion, an altered peritoneal environment, diminished ovarian reserve, and poorer oocyte quality [6,96,97]. For CE, in contrast, evidence for effects extending beyond the endometrium is much less developed. Persistent endometrial inflammation could still influence reproductive function indirectly through abnormal tissue repair, vascular abnormalities, and ongoing inflammatory signaling [29,35], but there is little direct evidence in women that CE impairs ovarian function or embryo competence.

For this reason, any additive or interactive effect between EM and CE is currently most convincing at the level of endometrial receptivity rather than across every aspect of fertility. CE may aggravate implantation defects already linked to EM, while EM may reduce the chance of successful conception through pelvic and ovarian pathways. This distinction is important because the coexistence of both conditions should not be assumed to impair ovarian reserve, oogenesis, sperm transport, embryo competence, and implantation to the same degree. What remains uncertain is whether the coexistence of the two conditions leads to poorer reproductive outcomes than either disorder alone, whether measured by natural conception, assisted reproductive technology outcomes, or miscarriage risk.

## 7. Future Directions

Interest in the coexistence of EM and CE has grown, but several fundamental issues remain unsettled. These include how CE should be defined diagnostically, how patients should be stratified by EM phenotype and CE severity, whether the relationship between the two conditions is causal or parallel, and whether integrated evaluation has meaningful clinical value for reproductive care. From a clinical perspective, current evidence does not support routine dual invasive evaluation for all women with infertility or universal CE screening in all patients with EM.

The current literature is not yet sufficient to answer these questions. Major limitations include heterogeneous study designs, infertility-enriched populations, the lack of standardized diagnostic criteria for CE, the small number of prospective studies examining the temporal relationship between EM and CE, inadequate phenotypic stratification, limited validation of multimodal diagnostic models, and the absence of robust data linking diagnosis and treatment to reproductive outcomes [9,11]. The main knowledge gaps and corresponding research priorities are summarized in Table 4.

Progress in this field will depend on more consistent diagnostic definitions, particularly standardized CE criteria including CD138 thresholds, biopsy timing, and specimen interpretation, prospective and stratified study designs, validation of integrated diagnostic approaches, and the use of clinically relevant reproductive endpoints. Future studies should also determine whether identifying and treating CE in women with EM improves implantation, miscarriage, assisted reproductive technology outcomes, or live birth. Only with this type of evidence will it be possible to judge whether coexisting EM and CE constitutes a clinically actionable reproductive phenotype or simply an association observed in selected populations.

## 8. Conclusions

In selected infertility populations, EM and CE may coexist more often than is generally appreciated, and this overlap may have clinical relevance. Although the two conditions arise in different anatomical locations, published studies suggest that they are linked by shared inflammatory, immune, and endometrial disturbances and may be found together more frequently in selected infertility cohorts [9,11,12,14,15,16]. At present, however, this association should be interpreted as hypothesis-generating rather than proof of a unified disease entity or a broadly synergistic reproductive disorder. The implications remain difficult to define, owing to methodological heterogeneity, probable selection bias, and the lack of standardized diagnostic criteria for CE [9,11]. At this stage, concurrent EM–CE evaluation should therefore be considered selectively rather than routinely, based on clinical phenotype and reproductive history. Further prospective, standardized, and stratified studies are needed to determine whether integrated diagnosis and management can improve reproductive outcomes.

## Figures and Tables

**Table 1:** Representative studies evaluating the epidemiological association between endometriosis and chronic endometritis.

Category	Study	Year	Study Design	Population/Sample	Main Finding	Main Limitation	Ref.
Direct coexistence and association studies	Takebayashi et al.	2014	Observational	Women undergoing gynecologic evaluation, including EM cases	Reported a significant association between EM and CE. CE in EM 52.9% vs. non-EM 27.0%	Early study; diagnostic criteria for CE differ from later reports	[15]
	Cicinelli et al.	2017	Case–control	Hysterectomy patients with/without EM (stage IV)	Found a higher prevalence of CE in women with EM, CE in EM 38.5% vs. 14.1%; OR 3.8.	Referral bias and heterogeneity in CE diagnosis may affect the strength of association	[16]
	Lin KY et al.	2020	Cohort (nationwide)	84,150 women (EM vs. non-EM)	Endometritis → EM HR 1.58 (95% CI 1.48–1.68)	ICD-9 based; no histologic confirmation of CE	[21]
	Liu Y et al.	2019	Case–control	Infertile women with/without CE	CE associated with reduced *Lactobacillus*; EM mentioned as comorbidity.	Main focus on microbiota, not specifically designed for EM-CE coexistence	[22]
Supportive studies	Qiao X et al.	2023	Cohort	Infertile women with minimal/mild EM (*n* = 201)	CE in 24.4%; CE independently reduced pregnancy (HR 0.58) and live birth rates	Infertility cohort only; not designed for prevalence estimation	[23]
	Gawron et al.	2025	Prospective cohort study	Women with peritoneal EM (*n* = 64)	Peritoneal EM associated with endometrial inflammatory profile (OR 3.43)	Small sample; supports biological overlap, not direct coexistence	[24]
	Holzer I et al.	2021	Prospective cohort	Infertile women undergoing hysteroscopy/laparoscopy (*n* = 100)	CE associated with tubal occlusion (OR 5.27); positive correlation with rASRM score	Pilot study; indirectly supports EM-CE link	[25]
Reviews	Kalaitzopoulos DR et al.	2025	Meta-analysis	6 studies (*n* = 900)	CE in EM pooled 28%; OR 2.07 (95% CI 1.11–3.84)	High heterogeneity (I^2^ = 43%); different diagnostic criteria	[14]
	Kitaya K & Yasuo T	2023	Narrative review	Summarizes commonalities in immunity, microbiota, treatment	EM and CE share immune and inflammatory features	No quantitative synthesis	[12]
	Pirtea P et al.	2021	Systematic review	Focus on RPL	CE in RPL 29.7%; treatment improves LBR	Not specifically designed for EM-CE coexistence	[11]

Abbreviations: CE, chronic endometritis; CI, confidence interval; EM, endometriosis; HPF, high-power field; HR, hazard ratio; LBR, live birth rate; OR, odds ratio; rASRM, revised American Society for Reproductive Medicine; RPL, recurrent pregnancy loss. Note: Direct EM–CE coexistence evidence is separated from indirect mechanistic support and narrative background. Reported associations should be interpreted cautiously because of heterogeneity in patient selection, infertility enrichment, and diagnostic criteria for CE.

**Table 2:** Shared pathophysiological features of endometriosis and chronic endometritis relevant to infertility.

Mechanistic Domain	Evidence in Endometriosis	Evidence in Chronic Endometritis	Shared Consequence	Reproductive Relevance
Chronic inflammatory signaling	Persistent inflammatory activation with increased IL-1β, IL-6, IL-8, TNF-α, and NF-κB-related signaling contributes to lesion survival, stromal-cell proliferation, angiogenesis, and tissue remodeling [28,30,31,32,33,37,38,39].	Sustained low-grade endometrial inflammation with increased IL-1β, IL-6, TNF-α, and NF-κB-related activation has been described in CE [29,34,35,36,40,41].	Persistent inflammatory amplification and chronic tissue injury	Impaired implantation, reduced endometrial receptivity, and infertility
Immune-cell dysfunction	Reduced NK-cell cytotoxicity, increased macrophage infiltration, and abnormalities in dendritic cells, B cells, and T-cell subsets support a dysregulated immune microenvironment in EM [46,47,48,49,50,51,52,53,54,55,56,57,58,59].	CE is associated with increased local immunoglobulin expression, altered NK-cell subsets, elevated macrophage and dendritic-cell populations, and shifts in T-helper-cell balance [10,60,61,62,63].	Disturbed immune homeostasis and persistent local inflammation	Abnormal embryo–endometrium interaction and impaired receptivity
Hormonal imbalance and progesterone resistance	Progesterone resistance and altered estrogen-dependent signaling are central features of EM-related endometrial dysfunction [64,65].	Persistent inflammation in CE may interfere with hormone-dependent endometrial maturation, and CE has been associated with delayed progesterone-dependent transformation and impaired endometrial receptivity [11,40,41].	Abnormal endometrial transformation and impaired hormonal responsiveness	Reduced receptivity and defective implantation timing
Defective decidualization and endometrial maturation	EM is associated with altered endometrial gene regulation and impaired decidualization [64,65,66].	CE has been associated with displacement of the window of implantation, defective decidualization, and delayed endometrial maturation [11,40,41].	Embryo–endometrium asynchrony	Implantation failure and early reproductive loss
Microbiota-related mucosal immune activation	Reproductive tract dysbiosis and mucosal immune disturbance have been proposed in EM, although current evidence remains limited and less consistent [27,68,69,70].	CE is more directly linked to microbial persistence, LPS-related inflammation, and altered vaginal microbiota, including reduced lactic-acid-producing bacteria [35,36,67].	Chronic mucosal inflammatory priming and endometrial immune disturbance	Persistent endometrial dysfunction and reduced implantation potential
Abnormal tissue repair and remodeling	EM is associated with lesion persistence, fibrosis, pelvic remodeling, and chronic inflammatory repair responses [28,30,31].	Repeated inflammatory injury in CE is associated with altered transcription of cytokines, growth factors, and apoptosis-related mediators involved in endometrial repair [40,41].	Altered tissue homeostasis and incomplete resolution of inflammation	Suboptimal reproductive microenvironment

Abbreviations: CE, chronic endometritis; EM, endometriosis; LPS, lipopolysaccharide; NF-κB, nuclear factor kappa B; NK, natural killer. Note: These shared features provide a biologically plausible basis for EM–CE coexistence, but their relative contribution to infertility and comorbidity remains incompletely defined. The domains listed here should not be interpreted as having equivalent evidentiary strength.

**Table 3:** Diagnostic approaches relevant to suspected EM–CE coexistence.

Diagnostic Modality	Target Disease	Current Status	Main Assessment and Clinical Use	Main Limitation	Role in Suspected EM–CE coexistence	Ref.
Clinical symptoms and reproductive history	Both	First-line triage	Assesses pelvic pain, dysmenorrhea, abnormal uterine bleeding, infertility, recurrent implantation failure, or recurrent pregnancy loss; readily available for initial clinical triage.	Manifestations are nonspecific and may overlap across diseases	Helps identify patients who may warrant selective evaluation for concurrent disease	[12,16,90,91]
TVUS	EM	Standard first-line	First-line imaging for ovarian endometrioma and some deep infiltrating lesions.	Limited sensitivity for superficial peritoneal EM; does not assess CE	Useful for pelvic disease burden, but cannot exclude concomitant endometrial inflammation	[71]
Pelvic MRI	EM	Selected adjunct	Assesses deep infiltrating disease and anatomical extent with high soft-tissue resolution.	Less useful for subtle or superficial disease; not a routine tool for all patients	Complements TVUS in selected women with suspected complex EM	[71,81]
Laparoscopy with histological confirmation	EM	Reference standard in selected cases	Allows direct pelvic visualization and histological confirmation in selected cases.	Invasive; costly; not suitable for universal infertility screening	Confirms EM but does not assess the uterine cavity unless additional procedures are performed	[72]
Hysteroscopy	CE	Selected CE assessment	Visualizes the uterine cavity and findings suggestive of CE, including micropolyps, hyperemia, and stromal edema.	Findings are not fully specific and remain operator-dependent	Raises suspicion of CE when pelvic findings alone do not explain reproductive dysfunction	[73,74,75,76]
Endometrial biopsy with CD138 immunohistochemistry	CE	Core CE confirmatory tool	Detects endometrial stromal plasma cells and is widely used to support CE diagnosis.	Diagnostic thresholds are inconsistent; focal disease and biopsy timing may affect detection	Core tool for targeted CE evaluation in women suspected of having concurrent disease	[74,75,76,77,78]
Histopathology without immunostaining	CE	Supportive only	Assesses routine inflammatory changes and possible plasma-cell-related abnormalities; broadly available.	Lower sensitivity and greater interpretive variability than CD138-based assessment	May provide supportive information but is less robust for standardized CE diagnosis	[75,77,78]
PET-based imaging and elastography	EM	Investigational/specialized adjunct	May assess selected deep lesions or tissue characteristics beyond routine imaging in specialized settings.	Limited clinical validation and small study populations	Promising adjuncts for difficult or selected cases, but not suitable for routine concurrent-disease screening	[81,82,83]
Circulating and microRNA-based biomarkers	EM	Early-validation/experimental	Assesses candidate blood-, tissue-, or saliva-based signatures as potentially less invasive diagnostic adjuncts.	Most candidates remain in the discovery or early validation phase	Promising adjuncts, but not yet ready for routine use in infertility-related comorbidity assessment	[79,80,84]
Microbiota-based testing and inflammatory biomarkers	CE	Exploratory adjunct	Assesses dysbiosis patterns, LPS, IL-6, or menstrual blood markers as possible adjunctive indicators of inflammation.	Lack of standardization and uncertain diagnostic thresholds	May support future recognition of persistent intrauterine inflammation in selected patients	[35,67,85]
Multimodal diagnostic models	Both	Early-validation integrated approach	Integrates symptoms, imaging, hysteroscopy, histopathology, and molecular data to improve diagnostic precision.	Still investigational; requires external validation	Relevant future direction for concurrent EM–CE assessment	[86,87]
Artificial intelligence-assisted imaging and prediction models	Both	Early-development future adjunct	Uses imaging or preoperative data for risk stratification in selected settings.	Evidence remains early and disease-specific; broader validation is required	Potential future adjunct for integrated assessment, but not ready for routine implementation	[88,89]

Abbreviations: CE, chronic endometritis; EM, endometriosis; MRI, magnetic resonance imaging; PET, positron emission tomography; TVUS, transvaginal ultrasound. Note: Diagnostic approaches are grouped according to their current clinical status and potential role in selected EM–CE assessment. Most tools remain disease-specific; multimodal and artificial intelligence-assisted approaches should currently be regarded as investigational rather than routine clinical tests.

**Table 4:** Key knowledge gaps and future research priorities in endometriosis–chronic endometritis comorbidity.

Knowledge Gap	Why It Matters	Current Limitation	Research Priority
Lack of standardized diagnostic criteria for CE	Prevents reliable prevalence estimates and valid comparison across studies	Variable CD138 thresholds, biopsy timing, sampling methods, and pathologic interpretation remain unresolved	Establish consensus diagnostic criteria for CE, including standardized histologic thresholds and specimen assessment
Limited prospective evidence on EM–CE coexistence	Prevents clear assessment of temporal sequence and causal direction	Most available data are retrospective or cross-sectional and largely derived from infertility-centered cohorts	Conduct prospective cohort studies specifically designed to evaluate EM–CE comorbidity
Inadequate stratification by disease phenotype and severity	Different subgroups may have different biological and clinical relevance	EM is often treated as a single disease entity, and CE is frequently analyzed as a binary diagnosis	Perform phenotype- and severity-stratified analyses according to EM subtype, disease stage, and CE severity
Limited integrated diagnostic models	Concurrent disease may be missed when diagnostic pathways remain compartmentalized	Imaging, hysteroscopy, pathology, and biomarkers are rarely evaluated together in the same framework	Develop and validate multimodal diagnostic approaches combining clinical, imaging, hysteroscopic, histopathologic, and molecular data
Uncertain reproductive impact of coexistence	Clinical relevance depends on reproductive outcomes rather than coexistence alone	Few studies directly evaluate implantation, miscarriage, ART outcomes, or live birth in women with confirmed EM–CE comorbidity	Prioritize outcome-oriented studies using clinically meaningful reproductive endpoints
Uncertain value of integrated management	It remains unclear whether identifying and treating CE in women with EM improves reproductive outcomes	Evidence on intervention, treatment sequencing, and subgroup benefit remains limited	Conduct prospective interventional studies in selected infertility populations to evaluate integrated diagnostic and treatment strategies

Abbreviations: ART, assisted reproductive technology; CE, chronic endometritis; EM, endometriosis. Note: Future work should move beyond descriptive coexistence and focus on diagnostic standardization, stratified study design, clinically meaningful reproductive endpoints, and evidence-based integrated management.

## Data Availability

No new data were created or analyzed in this study. Data sharing is not applicable to this article.

## Abbreviations

The following abbreviations are used in this manuscript:

ART	Assisted reproductive technology
CD138	Syndecan-1
CE	Chronic endometritis
CI	Confidence interval
COX-2	Cyclooxygenase 2
CXCL13	C-X-C motif chemokine ligand 13
DC	Dendritic cell
EM	Endometriosis
ER	Endoplasmic reticulum
HPF	High-power field
HR	Hazard ratio
IKK	IκB kinase
IL	Interleukin
IL-1β	Interleukin-1 beta
IL-6	Interleukin-6
IL-8	Interleukin-8
iNOS	Inducible nitric oxide synthase
LBR	Live birth rate
LPS	Lipopolysaccharide
MMP	Matrix metalloproteinase
MRI	Magnetic resonance imaging
NF-κB	Nuclear factor kappa-B
NK	Natural killer
NLRP3	NLR family pyrin domain-containing 3
OR	Odds ratio
PET	Positron emission tomography
rASRM	Revised American Society for Reproductive Medicine
RIF	Recurrent implantation failure
RPL	Recurrent pregnancy loss
TLR4	Toll-like receptor 4
TNF-α	Tumor necrosis factor alpha
TVUS	Transvaginal ultrasound
VEGF	Vascular endothelial growth factor
WOI	Window of implantation
